# Double heterozygous *PSEN2/SLC20A2* mutations causing late‐onset Alzheimer's disease and primary familial brain calcification

**DOI:** 10.1002/alz70855_106054

**Published:** 2025-12-24

**Authors:** Joong‐Seok Kim, Hyuk‐je Lee, Bora Yoon

**Affiliations:** ^1^ The Catholic University of Korea, Seoul, NA, Korea, Republic of (South)

## Abstract

**Background:**

Autosomal dominant Alzheimer's disease (ADAD) and primary familial brain calcification (PFBC) are rare causes of cognitive decline. We analyzed the clinical and radiological findings of a 75‐year‐old man presenting with memory impairment, whose brain imaging revealed bilateral basal ganglia calcification, severe white matter hyperintensities, and significant amyloid deposition.

**Method:**

Neuroimaging included ^18^F‐Florbetaben PET/CT to assess amyloid deposition and MRI to evaluate white matter hyperintensities and cerebral microbleeds, with findings analyzed using standardized scales. A neurologist, blinded to clinical data, assessed imaging results, while PET SUVR values were converted to the Centiloid scale for comparison. Next‐generation sequencing of 1,097 dementia‐related genes was performed using high‐throughput sequencing and bioinformatics tools to identify potential genetic variants.

**Result:**

Neuroimaging revealed significant amyloid plaque deposition (SUVR 1.82, Centiloid 98.3) and dense mineral deposits in multiple brain regions, suggesting possible PFBC or hyperparathyroidism. MRI showed medial temporal lobe atrophy (grade 2), severe white matter hyperintensities, and five lobar cerebral microbleeds. Genetic analysis identified a likely pathogenic SLC20A2 mutation (c.1711G>A) and a PSEN2 variant of unknown significance (c.166G>A), potentially linked to disease.

**Conclusion:**

This is the first reported case of a *PSEN2* and *SLC20A2* double mutation in the literature.

